# Monocyte-derived macrophage assisted breast cancer cell invasion as a personalized, predictive metric to score metastatic risk

**DOI:** 10.1038/srep13855

**Published:** 2015-09-09

**Authors:** Keon-Young Park, Gande Li, Manu O. Platt

**Affiliations:** 1 Wallace H. Coulter Department of Biomedical Engineering, Georgia Institute of Technology and Emory University, Atlanta, GA 30332.

## Abstract

Patient-to-patient variability in breast cancer progression complicates clinical treatment decisions. Of women undergoing prophylactic mastectomies, many may not have progressed to indolent forms of disease and could have benefited from milder, localized therapy. Tumor associated macrophages contribute significantly to tumor invasion and metastasis, with cysteine cathepsin proteases as important contributors. Here, a method is demonstrated by which variability in macrophage expression of cysteine cathepsins, their inhibitor cystatin C, and kinase activation can be used to train a multivariate model and score patients for invasion risk. These enzymatic profiles were used to predict macrophage-assisted MCF-7 breast cancer cell invasion in the trained computational model. To test these predictions, *a priori*, signals from monocytes isolated from women undergoing mastectomies were input to score their cancer invasion potential in a patient-specific manner, and successfully predicted that patient monocytes with highest predicted invasion indices matched those with more invasive initial diagnoses of the nine patients tested. Together this establishes proof-of-principle that personalized information acquired from minimally invasive blood draws may provide useful information to inform oncologists and patients of invasive/metastatic risk, helping to make decisions regarding radical mastectomy or milder, conservative treatments to save patients from hardship and surgical recovery.

About one in eight women in the United States will develop metastatic breast cancer over the course of her lifetime^1^. However, patient-to-patient variability in disease progression continues to complicate clinical decisions in diagnosis and treatment for breast cancer patients^2^,^3^,^4^,^5^,^6^,^7^,^8^. Advancement in diagnostic and imaging techniques led to increases in early detection of breast cancer. This increase, however, often leads to premature and aggressive treatment of non-malignant lesions due to inherent uncertainty in malignant progression of the cancer. It has been reported in a meta-analysis that many women undergoing mastectomies may not have advanced to the indolent form of the disease^9^, but currently there are not reliable ways of predicting whom would be at greatest risk and benefit the most from the aggressive course of therapy. A lack of well-informed risk/benefit analysis can result in net harm to the patients. Together, this highlights the importance and need for targeted, personalized treatments^10^ in the hopes of developing patient prognosis classification criteria based on biochemical analyses conducted with specimens obtained from a blood draw.

One central player promoting invasiveness of cancer cells is the tumor-associated macrophage (TAM)^11^. TAMs differentiate from circulating monocytes that leave the vasculature and enter the tumor tissue in response to a variety of cues secreted from the tumor and also in response to tumor microenvironment. Once there, they have been shown to promote angiogenesis^12^,^13^,^14^,^15^,^16^,^17^, tumor growth^13^, invasion and metastasis^18^ through secretion of cytokines to coordinate tumor-promoting immune responses^19^,^20^,^21^,^22^ as well as through secretion of tissue-remodeling cysteine cathepsin proteases^23^,^24^,^25^,^26^,^27^. Moreover, infiltration of TAMs is often associated with poor prognosis^28^,^29^,^30^, and in more advanced tumors, TAMs may make up as much as 50% of tumor volume^23^,^31^.

Cathepsins and their endogenous inhibitors, the cystatins, are secreted by TAMs have been shown to play significant role in cancer growth and invasion^32^,^33^,^34^. Cysteine cathepsins are proteases that have been identified as the most potent mammalian collagenases and elastases that, upon secretion, locally degrade extracellular matrix substrates^35^,^36^. Cathepsins K, L, S, and V produced by macrophages or by the cancerous cells themselves are highly implicated in tumor associated tissue remodeling and metastasis^37^,^38^,^39^,^40^,^41^,^42^,^43^, and there have been major efforts to develop pharmacological inhibitors to block their activity^44^,^45^,^46^.

Previously, we showed person-to-person variability in cathepsin activity from primary monocyte-derived macrophages and osteoclasts^47^. We were able to train a partial least squares regression (PLSR) model that could predict, *a priori*, the levels of cathepsins K, L, S, and V from monocyte-derived macrophages (MDMs) with greater than 90% predictability, using kinase activation signals quantified during monocyte differentiation^47^. For this study, those tools were applied to a cancer disease context using an *in vitro* system with freshly isolated monocytes to identify kinase signals that contribute most significantly toward proteolytic invasion outcomes, and to *a priori* classify patient MDMs by their invasion assisting potential in a more personalized manner. We then went on to identify proteolytic or molecular signatures in circulating, undifferentiated monocytes that could be useful for identifying patients at greatest risk for macrophage-mediated tumor cell invasion and increased metastatic potential. Our hypothesis is that through proteolytic profiling of monocytes obtained through a blood draw, and coupling this with a manageable multivariate phosphokinome analysis trained on previous patients’ data, that a patient’s risk for macrophage assisted tumor cell invasion could be predicted. Understanding the relationships between phosphokinase signaling, proteolytic activity, and breast cancer disease progression will allow for earlier predictions of indolent disease from a blood draw, and provide important clinical information to provide the patient and the oncologist additional personalized information to decide aggressive vs. more localized treatment.

## Results

### Person-to-person variability in monocyte-derived macrophage cathepsin activity and cystatin C level are related to variability in macrophage-assisted cancer cell invasion

**Figure 1:**
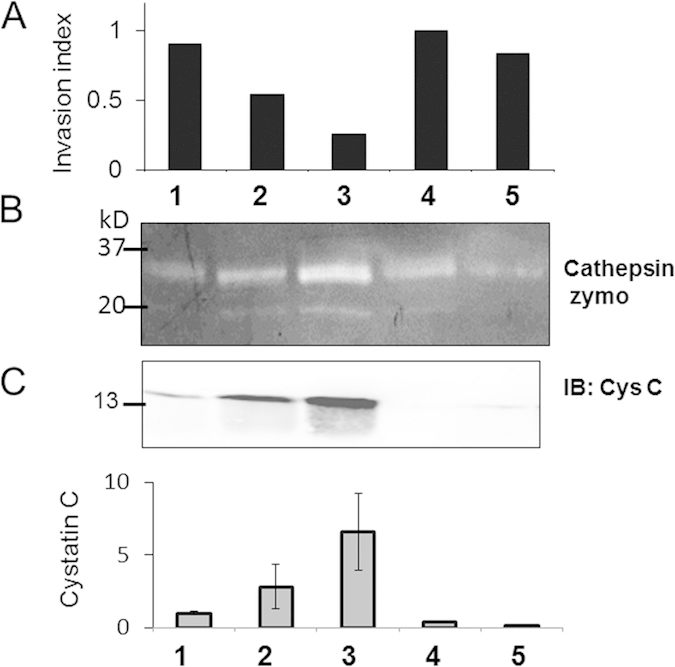
Cathepsin to cystatin C ratio influences patient-to-patient variability in cancer cell invasion. (**A**) Monocytes isolated from five patients among the first set were stimulated with M-CSF for 14 days to differentiate them into macrophages. On day 14, differentiated macrophages were plated onto a transwell coated with Matrigel with or without MCF-7 breast cancer cells, and allowed to transmigrate through to the other side. After 24 hours, number of invaded breast cancer cells were counted and invasion index was calculated by dividing the number of MCF-7 cells that invaded through the Matrigel and transwell when co-cultured with MDMs by the number of MCF-7 cells that invaded in the absence of MCF-7 cells. (**B**) Conditioned media was collected from days 14-15 and loaded for cathepsin zymography to measure secreted cathepsin activity which was quantified through densitometry. Zymogram gels and blots are cropped for space in the figure (**C**) The amount of cystatin C in conditioned media was measured using Western blotting with densitometry shown in the graph below.

Tumor-associated macrophages contribute to tumor cell invasion, and active cathepsins released by these macrophages have been shown to play significant contributing roles^23^,^24^,^25^,^26^,^27^. After previously demonstrating a wide range of inherent variability in cathepsin activity profiles of monocyte-derived macrophages, we first tested here the hypothesis that person-to-person variability in macrophage cathepsin activity would be reflected in macrophage-mediated cancer cell invasion. MCF-7 cells were chosen as they are estrogen-receptor positive which comprise the highest percentage of breast cancer in the U.S., and they are commonly used for research studies; this cell line was co-cultured with monocyte-derived macrophages, differentiated from freshly isolated monocytes. Buffy coats were isolated from peripheral blood drawn from healthy persons on a Ficoll gradient, monocytes were isolated by cell adhesion, and they were then differentiated into macrophages with M-CSF for 14 days. On day 14, monocyte-derived macrophages (MDMs) were lifted and re-plated onto a transwell filter coated with Matrigel with or without MCF-7 breast cancer cells for modified Boyden chamber assay. After 24 hours, the number of cells that invaded through the membrane was counted, and an invasion index was calculated. The index was defined as the ratio of the number of invaded cells in the MCF-7/MDM co-culture system to the number of invaded cells in MCF-7-only culture; MCF-7s exhibited minimal invasion when cultured alone. These values were normalized between 0 and 1, with 1 being the highest invasion index (patient 4), and of the five unique persons tested, variability was 0.3 or 30% increased invasion in MDM/MCF-7 co-cultures over that of the MCF-7 cells alone (Fig. 1A). Cancer cells co-cultured with patient 3 macrophages had the least invasion index which was 25% of patient 4 and 50% of patient 2.

To determine if there was a correlation between amounts of cathepsins secreted by monocyte-derived macrophages and the invasion index, conditioned media was collected from differentiated macrophages after 14 days and equal amounts of protein were loaded for multiplex cathepsin zymography (Fig. 1B). Whereas patient 4 had the highest macrophage mediated cancer cell invasion, patient 3 had the highest cathepsin activity as indicated by the zymogram (Fig. 1B), but the lowest invasion among those tested here (Fig. 1A). To explain this discrepancy, we determined variability in expression of cystatin C, the endogenous protein inhibitor of the cathepsin family of proteases, that has been shown to be secreted by monocyte-derived macrophages^48^, and a lack of cystatin C has been shown to promote cancer cell invasion^49^. We quantified cystatin C levels from the conditioned media using Western blot and found that person 3 with highest cathepsin activity and lowest invasion also had highest cystatin C levels in the conditioned media (Fig. 1C).

**Figure 2:**
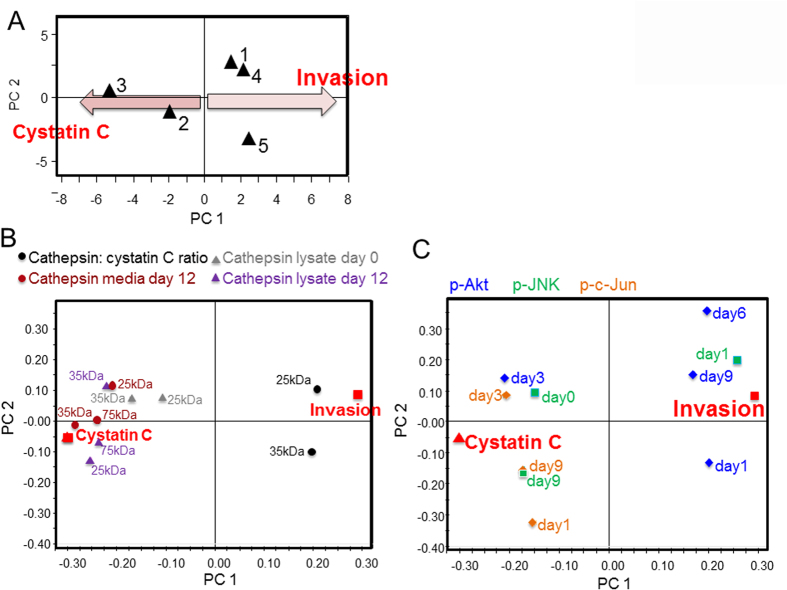
Kinase profiling and PLSR analysis implicate JNK/c-jun and Akt in macrophage-mediated cell invasion. PLSR models were built with inputs from these five persons after kinase phosphorylation measured using Bioplex® bead kits on days 0, 1, 3, 6 and 9 of differentiating cells from freshly isolated monocytes using M-CSF. Other inputs were cathepsin activity of monocyte-derived macrophages at day 12. Outputs included were macrophage-assisted breast cancer cell invasion and cystatin C levels as determined by ELISA. (**A**) Scores plot separates the patient along the principal components of invasion vs. cystatin C expression. (**B**) Loadings plots shows co-variance among the cathepsin signal measurements with the outcomes of invasion or cystatin C. Calculated metrics of secreted cathepsin-to-cystatin ratio for each person were also input and clustered closely with the invasion response. (**C**) Loadings plot showing co-variance of the phosphokinase signatures with the outcomes of invasion or cystatin C. These are plotted separately for easier visual representation.

To determine the factors that contributed to macrophage- and cathepsin-mediated invasion, we applied partial least square regression (PLSR) analysis, a statistical method that establishes a mathematical relationship between cue, signals, and responses, cues here being patient-specific differences due to genetics and preconditioning of monocytes prior to isolation from the blood that cannot separately be quantified, but impact downstream kinase activation signatures. Scores plot in the PLSR model plotted the patients based on their kinase signatures, cathepsin activity, and cystatin C expression in principal component space (Fig. 2A). The loadings plots were used to show co-variance among the signal measurements with the outcomes of invasion vs. cystatin C (Fig. 2B,C). The calculated metrics of secreted cathepsin-to-cystatin ratio for each person were also input, and it was determined that the cathepsin-to-cystatin ratio clustered closely with the invasion response among these persons (Fig. 2B), even more than individual cathepsin activity of several different cathepsin family members (25, 35 or 75 kDa sizes) whether measured from the cell lysate (purple or gray triangles) or secreted into the conditioned media (brown circles). Together, these data indicate that the first principal component defines the invasion vs. cystatin C inhibition axis,

### Profiling kinase phosphorylation implicates JNK/c-jun and Akt in macrophage-assisted cancer cell invasion

Since we previously determined that kinase activation signatures were predictive of cathepsin expression of the differentiated MDMs^47^, kinase activation signals were collected during the 12 day period of the monocytes differentiating into macrophages. In order to identify kinase signals that contribute most significantly toward proteolytic and invasion outcomes, as well as provide a rationale behind differences among the patients tested, lysates from differentiating monocytes on days 1, 3, 6 and 9 and phosphorylation of six kinases were measured with Bioplex: ERK1/2, Akt, p38 MAPK, JNK, and c-Jun, as done previously by us^47^. These signals were included in the same PLSR model as the cathepsin signatures, but plotted on a separate graph for clarity in the loading plot (Fig. 2C). p38 and ERK1/2 activation did not show significant differences across treatments for principal component 1 or 2 so are not shown in the plot. After calculating variable importance of projection (VIP) scores, a metric that identifies which signals contribute greatest to the response, JNK/c-Jun phosphorylation and Akt phosphorylation were ranked highest, and also co-varied strongly with the responses of invasion or cystatin C (Fig. 2C). A strongly negative projection also suggests an inverse correlation to the particular response, such that c-jun and JNK signals that are clustered around cystatin C could also indicate that their reduced phosphorylation could promote that response.

### Variability in monocyte-derived macrophage cathepsin activity, cystatin C level, and assisted invasion

**Figure 3:**
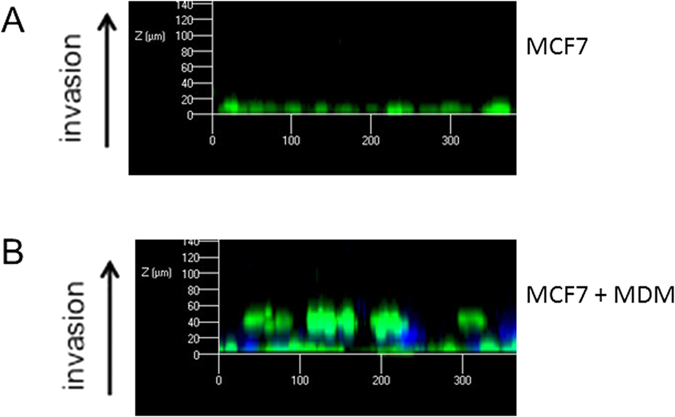
MDMs assist MCF-7 invasion through collagen gels. After differentiating monocytes for 12 days, monocyte-derived macrophages were labeled with CellTracker blue then co-cultured with GFP-expressing MCF-7 breast cancer cells and allowed to invade through a collagen I gel. Confocal microscope was used to image the height of the gel and quantify the number of cells that invaded past 30 μm. (**A**) GFP-expressing MCF7 cells do not significantly invade into the collagen I gel without the MDMs, but (**B**) co-culture with the MDMs increases the invasion of the GFP-tagged MCF-7 cells.

We replaced the Boyden chamber invasion assay used earlier with a fluorescent tracking assay to distinguish monocyte-derived macrophages from GFP-labeled MCF-7 cells. Macrophages were stained with CellTracker Blue and re-plated with MCF-7 breast cancer cells stably expressing green-fluorescent protein, then overlaid with 2.5 mg/ml collagen I gels with medium. After 24 hours, the collagen discs were fixed and breast cancer cells invading at least 20 μm into the collagen were counted by taking 5 μm optical z-slices with confocal microscopy (Fig. 3). Here, the invasion index was defined as percentage of the breast cancer cells that invaded beyond 20 μm among the total number of breast cancer cells.

**Figure 4:**
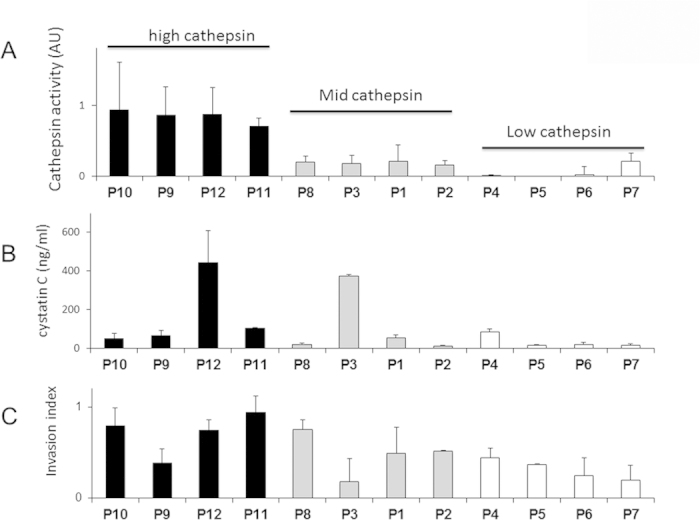
Variability in monocyte-derived macrophage cathepsin activity, cystatin C level, and assisted invasion. Freshly isolated monocytes from the previous 5 persons plus 7 others were differentiated for 13 days. On day 14, differentiation media was replaced with serum free RPMI and lysates and conditioned media were collected after incubating cells for 14-16 hours. (**A**) Cathepsin activity was measured using multiplex cathepsin zymography, (**B**) cystatin C level was measured with ELISA, and (**C**) invasion was determined as described in Fig. 3 and Methods. For those tested, they could be grouped into high, mid, and low cathepsin producers, and there is no correlation between cathepsins produced and cystatin C produced.

Freshly isolated monocytes from 12 patients (7 new patients recruited into study) were differentiated into macrophages for 14 days. Using cathepsin zymography on the conditioned media, this set of macrophages could be grouped into high (black), mid (gray), or low (white) cathepsin producers when quantifying secreted active cathepsins (Fig. 4A), again demonstrating variability among different individuals in their cathepsins produced. Secreted cystatin C levels were also measured from conditioned medium by ELISA, but there was no correlation between secreted cathepsin activity and secreted cystatin C levels across the patients (Fig. 4B), and while there was a trend towards the higher and mid cathepsin producers having a higher invasion index than the low cathepsin producers (Fig. 4C), again there was no statistically significant correlation.

### Predictions of macrophage-assisted invasion using kinase signatures and cathepsin activity of freshly isolated monocytes

**Figure 5:**
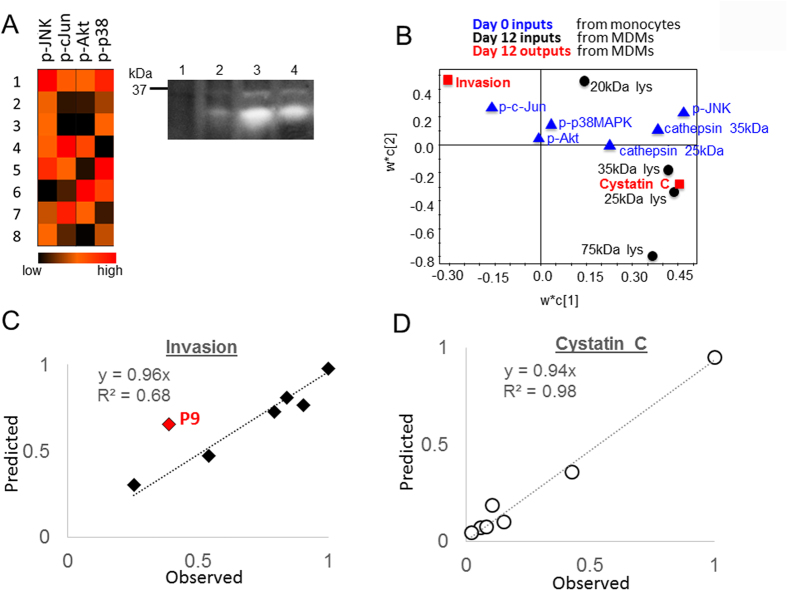
Phosphokinase and cathepsin signatures of freshly isolated monocytes can be used to predict macrophage-assisted invasion phenotype. (**A**) Monocytes isolated freshly from whole blood were lysed for phosphokinase analysis as well as cathepsin activity by zymography. Representative zymogram is shown for four patients and is cropped for space, but shows all cathepsin active bands per patient. (**B**) A new PLSR model was generated trained solely on day 0 kinase activation (blue) as well as day 12 cathepsin activity of lysed differentiated macrophages of patient 1, 2, 3, 4, 5, 9, and 10 (black), with Y-block responses of cystatin C and invasion (red). The loadings plot is shown with day 0 inputs labeled blue, day 12 inputs black, and day 12 outputs red. (**C**) Invasion was predictive for most patients but outlier patient# 9 reduced predictability to 68% overall. (**D**) However, cystatin C levels were highly predictive 98%.

Having trained the model on the ultimate invasion assistance potential of the differentiated macrophages as a model of the TAMs, we next determined if the macrophage invasion and cystatin C profile could be predicted from signatures measured exclusively from freshly isolated monocytes. To do this, however, we would train the model based on the monocyte-differentiated macrophage data from these matching patients and test predictability of the day 0 signals alone to predict the outcomes of invasion and cystatin C production using bootstrapping of the input data. Monocytes were isolated from whole blood of these same persons using magnetic activated cell sorting with anti-CD14 beads, and then lysed immediately to measure JNK, c-jun, Akt, and p38 phosphorylation. A heat map of the normalized kinase phosphorylation signatures from day 0 monocytes is shown in Fig. 5A for 8 persons. Variability in baseline monocyte kinase phosphorylation signatures for those tested is obvious indicating the inherent variability even of the freshly isolated cells. Cathepsin activity was also measured from the freshly isolated monocytes by zymography with a representative zymogram shown for patients 1–4, again with person-to-person variability in the intensity of cathepsin activity across these specimens (Fig. 5A).

The new PLSR model was trained based with input signals of the monocyte day 0 kinase activation signals for JNK, c-jun, and p38, day 12 cathepsin activity from cell lysates of the monocyte-derived macrophages from these same persons as collected in the earlier experiments. Response matrix was macrophage-assisted invasion and cystatin C measured from the earlier experiments, and the established relationship for the weighted coefficient matrix calculation was that day 0 signals could be used to predict 12 day invasion and cystatin C outcomes, measured from the earlier study of the monocyte-derived macrophages. The loadings plot of this PLSR model is shown in Fig. 5B showing the separation of the two outcomes of invasion and cystatin C in red in principle component space, as well as plotting the day 0 signals from monocytes in blue compared to the day 12 signals from the macrophages in black. This new model was first tested for predictability of macrophage assisted invasion based on day 0 inputs and it was 68% (Fig. 5C), with one patient (P9) as a potential outlier lowering the predictability, but was 98% predictive for cystatin C that would be produced by the differentiated macrophages (Fig. 5D). If P9 was excluded from the analysis, then the predicted invasion would increase to 95%.

### Predicting prognosis for women undergoing surgery for breast cancer using monocyte phosphokinase and cathepsin signatures

**Figure 6:**
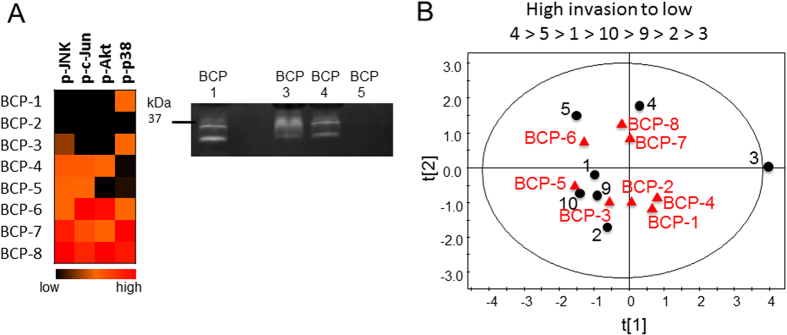
Phosphokinase and cathepsin signatures of freshly isolated monocytes from breast cancer patients were predicted using model trained on macrophage-assisted invasion. Whole blood was collected from breast cancer patients (BCP) undergoing surgery (lumpectomy, mastectomy), and monocytes were freshly isolated and lysed for (**A**) phosphokinase signatures and cathepsin activity with a representative cathepsin zymogram shown for four patients and cropped for space, but shows all cathepsin active bands per patient. These data were then input into the trained PLSR model to calculate the outcomes of the monocyte-derived macrophage-assisted invasion and cystatin C levels after differentiation to determine if blood signatures would be useful to predict patient invasion outcomes. (**B**) The loadings plot is shown and unique breast cancer patients co-vary with patients from the trained model with highest invasion of #4 down to #3 with lowest invasion to indicate possible phenotypes of the MDMs from the women with breast cancer.

Ultimately, the goal was to be able to apply these predictions obtained from monocytes collected from a blood draw, towards women diagnosed with breast cancer to inform clinical decisions for surgery and treatment amid the patient-to-patient variation. To test if phosphokinase and cathepsin signatures from monocytes isolated from a simple blood draw could be predictive of macrophage-mediated cancer invasion potential, we tested this approach on monocytes obtained from whole blood of women undergoing partial, full, or double mastectomies. Monocytes were lysed without differentiation for phosphokinase analysis by Bioplex and cathepsin activity by zymography (Fig. 6A). These data were included in the X matrix of the PLSR model trained with the day 0 kinase and cathepsin data, input into the previously trained model with full phosphokinase and cathepsin data sets used in Fig. 6 with high predictability (except for the outlier patient 9). Day 0 values for phosphorylation of JNK, c-jun, Akt, and p38 were determined as well as cathepsin activity from the cell lysates (Fig. 6A), just as was done with the monocytes used to train the model. The weighted coefficient matrix calculated from the trained model was used to predict invasion and cystatin C responses of the monocytes from the breast cancer patients if they were differentiated into monocyte-derived macrophages over the 12 day period. A new scores plot was generated from this predictive model to cluster breast cancer patients with the well-defined 7 person trainers as an indicator of phenotype (Fig. 6B).

The predicted values of monocyte-derived macrophage assisted invasion and cystatin C levels from the day 0 breast cancer patients (BCP#) were then ranked in order of lowest to highest cystatin C levels (Table 1) or by invasion index (Table 2), with their ranks interspersed with the actual values from the known, tested MDM data used to train the model. Cystatin C was used to rank first since it had higher predictability than invasion (Fig. 5). When matched with patient initial diagnoses, two out of the top three breast cancer patients with the lowest predicted cystatin C levels were those with invasive lobular carcinoma (Table 1). Whether ranked by cystatin C levels or invasion index (Table 2), breast cancer patients (BCP) 5, 6, 7, and 8 are consistently scored higher than the others, and their initial cancer diagnoses were invasive lobular, bilateral-invasive lobular, and intraductal carcinomas (IDC).

## Discussion

In this study, we showed that interpersonal variability in macrophage-assisted cancer cell invasion is associated with patient variability in macrophage cathepsin activity and cystatin C level. Tumor associated macrophages are emerging as a central player in promoting tumor growth, and our results suggest that variability in macrophage cathepsin and cystatin C level could sufficiently confer interpersonal variability in cancer cell invasiveness, and at a minimum may provide predictive information to help guide clinician/patient decisions regarding treatment. In our disease model system, cancer cells were obtained from a clonal source, MCF-7s, whereas the macrophages were patient-specific and differentiated from monocytes isolated from individual patients, but differentiated under similar conditions. Therefore, it may be that an important contribution to the variability in breast cancer cell invasion and tumor growth and spread may be due to the TAMs that infiltrate, and locally secrete cathepsins and cystatin C that can directly assist cancer cell invasion. As a predictive metric, this holds promise by providing insight into the importance of biomarkers in the local tumor microenvironment as opposed to studies that have sought metastatic correlation after serum measurement of cathepsins or cystatin C^51^,^52^,^53^. Cathepsins in serum may be from a number of sources, including cardiovascular sources for which cathepsin levels have been correlated with atherosclerosis, diabetes, and abdominal aortic aneurysms^54^,^55^.

We showed that there is patient-to-patient variability in macrophage cathepsin activity and secreted cystatin C level and that variability is associated with interpersonal variability in macrophage-mediated cancer cell invasion (Fig. 1). Taken together, these results highlight the importance of patient-specific profiling of disease potential and intervention as well as the potential utility of using circulating monocytes and macrophages as predictive markers for breast cancer progression.

Although cystatin C is thought to be expressed constitutively by most cells, studies have shown changes in its expression in disease states^56^,^57^,^58^ and in different cell subtypes^59^. In addition, there are two functional polymorphisms in the promoter region of cystatin C gene, which showed association with higher disease incidence^60^. We showed that cystatin C protein level secreted by macrophages varies between individual patients and that this may play a role in modulating invasive potential, in part by changing the amount of active cathepsins available to degrade the surrounding matrix. Cathepsins secreted by tumor-associated macrophages promote cancer cell invasion^61^, and the balance between cathepsins and cystatin C secreted by cancer cells is an important determinant of tumor grade^62^. Although cathepsins and cystatin expression level and activity of breast cancer cell subtypes are not well understood, studies have shown that subtypes have distinct metastatic site preference, recurrence rate, immune responses and invasive potential^63^,^64^,^65^. As it has been shown that inhibition of specific cathepsins reduces breast cancer bone metastasis^66^,^67^, it would be reasonable to conjecture that distinct level of cathepsins and their inhibitors produced by breast cancer subtypes and tumor associated macrophages attribute to cancer invasive potential and metastasis. The techniques tested in this study may be able to translate these measurements into prognostic indicators for patients to inform aggressive surgical decisions.

We found no apparent correlation or feedback between secreted cathepsin and cystatin C (Fig. 4), although there was a wide range of variability among the cohort gathered here. Signaling pathways that regulate cathepsin or cystatin C expression and feedback loops between the two are not yet defined. Further work is necessary to investigate whether cells actively modulate the amount of secreted cystatin C to compete with extracellular cathepsins available to degrade ECM and assist cancer cell invasion. Understanding this mechanism would also be important for proper dosing of the cathepsin inhibitors which have been suggested for adjuvant therapy for cancer and other tissue-remodeling diseases^68^,^69^,^70^,^71^,^72^.

As more patient data and patient outcomes are collected in continual validation and training of this model, predictions can begin to include recurrence of breast cancer as another indicator of metastatic potential to be predicted from earliest information from a minimally invasive blood draw. It was important here to use a clonal breast cancer cell line co-cultured with the patient-derived macrophages to distinguish the MDM contribution from the breast cancer differences to demonstrate the role MDMs, as a surrogate for TAMs, play in metastatic disease. Variability in patient tumor cell genetics, aggression, and migration is also probably an important contribution to the metastatic ability, but these genetic instabilities cannot be predicted ahead of sampling the tumor cells. We propose here that inherent invasive ability of the monocyte-derived macrophages via their production of proteases exists within a range of values, which we have shown previously^47^ and in these studies (Fig. 4), may be able to distinguish and score patients’ speed of progression and prognosis.

Our underlying assumption in the interpretation of this scoring of patients based on predicted cystatin C levels and tumor invasion involves patient presentation of symptoms. The assumption used is that after patients presented via self-exam, mammography, or other symptoms that they waited equal times prior to visiting the doctor and confirming diagnosis. Then we assumed that those initially diagnosed with more progressed tumors was an indication of a tumor that advanced faster than others, although this may not be the case. More advanced patients may have just discovered a lump or went to a doctor at a later time than others. However, the stratification of macrophage-assisted invasion in the non-cancer patients indicates that these ranges of activity are inherent and would be different among the cancer presenting patients as well.

In summary, this study is a proof-of-principle of this novel technique to possibly predict breast cancer prognosis, but is not yet sufficiently validated for application to current breast cancer patients. The predictive model should be validated with longer term patient outcomes of 5 year survival rate, recurrence, or metastasis, which would improve its predictive power with this more robust dataset. By adding a greater number of patients to this compendium of data and accumulate more cathepsin and kinase profiles to train the model, threshold scores for cystatin C and invasion may be determined that will define a patient as clearly high risk and warranting mastectomy compared to a lower value that could indicate local radiation or lumpectomy.

## Materials and Methods

### Primary monocyte isolation and differentiation

Heparinized venous blood from healthy volunteers was diluted 1:1 in sterile PBS and layered on Ficoll-Paque (GE healthcare) and centrifuged at 400 g for 30 minutes. The buffy coat layer was isolated, red blood cells lysed with red blood cell lysis buffer (0.83% ammonium chloride, 0.1% potassium bicarbonate, and 0.0037% EDTA), and peripheral blood mononuclear cells (PBMCs) were washed 3 times in PBS. For monocyte phosphoprotein and cathepsin activity analysis, CD14^+^ and CD16^+^ monocytes were isolated using Pan-monocyte magnetic bead isolation kit (Miltenyi Biotec) and lysates were collected. For macrophage differentiation, monocytes adhered overnight on tissue-culture plates (Corning), were cultured in RPMI medium 1640 (Mediatech) containing 10% fetal bovine serum (FBS, Atlanta Biologicals), 1% L-glutamine, and 1% penicillin/streptomycin (Life Technologies) 10% male human serum and 30 ng/μl macrophage colony stimulating factor (M-CSF, Peprotech). Medium was replaced every 3 days. All human studies were approved by Institutional Review Boards at Georgia Institute of Technology and Dekalb Medical Center. Informed consent was obtained from all subjects and methods were carried out in accordance with approved guidelines.

### Multiplex cathepsin zymography

Cell extracts and conditioned media from monocytes or macrophages were collected. To prepare conditioned media, differentiation media was replaced with serum-free media on day 14 and incubated overnight. Conditioned media was collected and concentrated using VivaSpin®500 Centrifugal Concentrator (Vivaproducts). Cellular protein was extracted in lysis buffer (20 nM Tris-HCl at pH 7.5, 5 mM EGTA, 150 mM NaCl, 20 mM β-glycerol-phosphate, 10 mM NaF, 1 mM sodium orthovanadate, 1% Triton X-100, 0.1% Tween-20) with 0.1 mM leupeptin freshly added. Cathepsin zymography was performed on cell extracts and on conditioned media as described previously^73^. Densitometry was performed using ImageJ to quantify the intensity of the white cleared band of proteolytic activity.

### Measurements of cystatin C

Conditioned media collected from differentiated macrophages were loaded for SDS-PAGE and Western blot on nitrocellulose membranes (Bio-Rad) using mouse polyclonal antibody against cystatin C (Santa Cruz Biotechnology), or with Quantikine Cystatin C ELISA kit (R&D Biosystems).

### Collagen invasion assay

Collagen invasion assay was adopted from previous work by Goswami *et al.*^11^ On day 13, MDMs were stained using 25 μM CellTracker Blue CMAC (Invitrogen) for 90 minutes. Then 160,000 MDMs and 64,000 MCF-7 cells were plated on a 12-well MatTek multiwell plates in RPMI with 10% human AB serum (Innovative Diagnostics). After overnight incubation, media was replaced with serum-free RPMI for four hours. Then cells were overlaid with 1 mm layer of 2.5 mg/mL collagen I and was allowed to gel for 90 minutes at 37 °C before adding 1 mL of RPMI with 10% human AB serum. After 24 hour incubation, cells and the collagen gels were fixed with 10% neutral buffered formalin and imaged by confocal microscopy. Optical z-sections were taken every 5 μm from the bottom of the plate. MCF-7 cells that invaded into the collagen gel beyond 20 μm were counted and were divided by the number of MCF-7 cells at the bottom of the plate.

### Kinase phosphorylation analysis

On days 0, 1, 3, 6 and 9, freshly isolated monocytes or differentiating cells stimulated with M-CSF were lysed and total protein was determined using BCA kit (Pierce). Bioplex® bead kits (BioRad) were used according to manufacturer’s instructions with 5 μg protein from each sample and measured phosphorylation of Akt (Ser^473^), p38 MAPK (Thr^180^/Tyr^182^), JNK (Thr^183^/Tyr^185^), c-jun (Ser^63^), IκB-α (Ser^32^/Ser^36^) and ERK1/2 (Thr^202^/Tyr^204^, Thr^185^/Tyr^187^). Signal values for each phosphorylated kinase were normalized to the signal detected in a master lysate prepared in bulk from pre-stimulated cells that was used as a control for all assays. Signal values for each kinase were normalized to between 0 and 1 by dividing by the maximum value over the entire 9 days for all treatments.

### Partial least square regression analysis (PLSR)

M × N data matrix was generated with data from M patients and N kinase phosphorylation signals or N cathepsin activity and cystatin C level. Each column of the independent X matrix corresponds to a unique input or signal: phosphorylated kinase signal from days 0, 1, 3, 6, and 9, and each column of the dependent Y matrix corresponds to unique responses which were cathepsin activity, cystatin C level or invasion index. Each row represents a unique patient from which the monocytes were isolated. All data was mean-centered and scaled to unit variance. SIMCA-P (UMetrics) was used to solve the PLSR problem with the nonlinear iterative partial least squares (NIPALS) algorithm.

## Additional Information

**How to cite this article**: Park, K.-Y. *et al.* Monocyte-derived macrophage assisted breast cancer cell invasion as a personalized, predictive metric to score metastatic risk. *Sci. Rep.* **5**, 13855; doi: 10.1038/srep13855 (2015).

## Figures and Tables

**Table 1:** Trained PLSR model based on monocyte phosphokinase signatures, MDMs, and macrophage-assisted invasion of MCF7 cells, ranks actual breast cancer patients by cystatin C level using only monocyte signatures as inputs.

	Cys C	Invasion	Dx*	STAGE	GRADE	SIZE
**BCP-6**	−0.02	0.88	IDC	I	2	0.5 cm
5	0.043	0.81				
**BCP-5**	0.052	0.75	Bilat. Inv Lobular	IV	2	7.0 mm; 5.0 cm
**BCP-8**	0.068	0.91	Invasive Lobular	IIA	2	9.0 cm
4	0.069	0.98				
10	0.076	0.73				
1	0.101	0.77				
**BCP-7**	0.122	0.86	IDC	I	2	1.0 cm
*9*	0.189	0.65				
**BCP-3**	0.268	0.6	IDC	I	2	5.0 mm, 2.0 mm × 2 foci
2	0.36	0.47				
**BCP-2**	0.379	0.53	DCIS	0	3	3.5 cm
**BCP-4**	0.493	0.48	IDC	IIA	1	2.5 cm
**BCP-1**	0.493	0.46	IDC	I	2	0.9 cm
3	0.953	0.3				

Monocytes isolated from whole blood of breast cancer patients undergoing lumpectomy or mastectomy were lysed for phosphokinase analysis and cathepsin zymography without differentiation into macrophages. This data was input into the trained PLSR model of non-cancer persons’ macrophage mediated invasion, cathepsin activity, and cystatin C levels, and used to predict breast cancer patient invasion and cystatin C with only the day 0 inputs of p-JNK, p-c-jun, p-p38, and p-Akt, and monocyte active cathepsin at 25 kDa and 35 kDa (only ones detected in undifferentiated monocytes).

**Table 2:** Actual breast cancer patient invasion potential predicted and ranked with only monocyte signatures as inputs. As in table 1, except ranked by invasion index.

	Cys C	Invasion	Dx*	STAGE	GRADE	SIZE
4	0.069	0.98				
BCP-8	0.068	0.91	Invasive Lobular	IIA	2	9.0 cm
BCP-6	−0.02	0.88	IDC	I	2	0.5 cm
BCP-7	0.122	0.86	IDC	I	2	1.0 cm
5	0.043	0.81				
1	0.101	0.77				
BCP-5	0.052	0.75	Bilat. Inv Lobular	IV	2	7.0 mm; 5.0 cm
10	0.076	0.73				
9	0.189	0.65				
BCP-3	0.268	0.6	IDC	I	2	5.0 mm, 2.0 mm ×, 2 foci
BCP-2	0.379	0.53	DCIS	0	3	3.5 cm
BCP-4	0.493	0.48	IDC	IIA	1	2.5 cm
2	0.36	0.47				
BCP-1	0.493	0.46	IDC	I	2	0.9 cm
3	0.953	0.3				
